# Neurotransmitters—Key Factors in Neurological and Neurodegenerative Disorders of the Central Nervous System

**DOI:** 10.3390/ijms23115954

**Published:** 2022-05-25

**Authors:** Raluca Ioana Teleanu, Adelina-Gabriela Niculescu, Eugenia Roza, Oana Vladâcenco, Alexandru Mihai Grumezescu, Daniel Mihai Teleanu

**Affiliations:** 1 Department of Pediatric Neurology, “Dr. Victor Gomoiu” Children’s Hospital, 022102 Bucharest, Romania; raluca.teleanu@umfcd.ro (R.I.T.); eugenia.roza@umfcd.ro (E.R.); oana-aurelia.vladacenco@drd.umfcd.ro (O.V.); 2 “Carol Davila” University of Medicine and Pharmacy, 020021 Bucharest, Romania; daniel.teleanu@umfcd.ro; 3 Department of Science and Engineering of Oxide Materials and Nanomaterials, Politehnica University of Bucharest, 011061 Bucharest, Romania; adelina.niculescu@upb.ro; 4 Research Institute of the University of Bucharest—ICUB, University of Bucharest, 050657 Bucharest, Romania; 5 Academy of Romanian Scientists, Ilfov No. 3, 050044 Bucharest, Romania

**Keywords:** neurotransmitters, neurological disorders, neurodegenerative disorders, neurotransmitters detection, neurotransmitters modulation

## Abstract

Neurotransmitters are molecules that amplify, transmit, and convert signals in cells, having an essential role in information transmission throughout the nervous system. Hundreds of such chemicals have been discovered in the last century, continuing to be identified and studied concerning their action on brain health. These substances have been observed to influence numerous functions, including emotions, thoughts, memories, learning, and movements. Thus, disturbances in neurotransmitters’ homeostasis started being correlated with a plethora of neurological and neurodegenerative disorders. In this respect, the present paper aims to describe the most important neurotransmitters, broadly classified into canonical (e.g., amino acids, monoamines, acetylcholine, purines, soluble gases, neuropeptides) and noncanonical neurotransmitters (e.g., exosomes, steroids, D-aspartic acid), and explain their link with some of the most relevant neurological conditions. Moreover, a brief overview of the recently developed neurotransmitters’ detection methods is offered, followed by several considerations on the modulation of these substances towards restoring homeostasis.

## 1. Introduction

The central nervous system (CNS) processes information from and delivers information to the peripheral nervous system through signal conduction from one neuron to another via synapses. Thus, through synaptic transmission (also known as neurotransmission), CNS can control smooth, skeletal, and cardiac muscles, bodily secretions, and organ functions [1].

An essential role in information transmission throughout the CNS and peripheral nervous system is played by neurotransmitters (NTs), which are endogenous chemical messengers that carry and amplify nerve-to-nerve signaling or signals between nerves and other cell types. These small molecules are crucial for communicating sensory, motor, and integrative neuronal messages, affecting many functions, such as emotions, thoughts, memories, movements, and sleep patterns. These chemicals play essential roles in the functioning of the brain, being fundamental regulators of neuronal growth, differentiation, and survival. Consequently, abnormal levels of NTs are reflected in dysregulation of brain functions, leading to various physical, psychotic, and neurodegenerative diseases [2,3,4,5,6,7].

In this context, a deeper understanding of NTs’ roles and actions is mandatory for better assessing some of the most severe neurological disorders. Therefore, this review aims to shed some light on the various known canonical and noncanonical NTs, emphasizing their involvement in different relevant diseases. Moreover, novel NT detection methods are presented, followed by several modulation strategies for treating associated neurological conditions.

## 2. Neurotransmitters

NTs consist of molecules that amplify, transmit, and convert signals in cells, being key players in brain functions, behavior, and cognition. More than 200 such chemical messengers have been identified since 1921, yet the exact number of NTs is unknown. This happens especially because new biomolecules that exhibit neuroactivity are continuing to be added to a growing list of NTs [3,8].

For a neuroactive compound to be classified as an NT, it must satisfy the following requirements: (i) be produced and released by the same neuron and stocked at the presynaptic terminal; (ii) induce a specific behavior in the postsynaptic neuron; (iii) its exogenous administration must produce the same effect; and (iv) its induced action on the postsynaptic cell can be stopped by a specific mechanism [9].

Thus, several types of NTs have been recognized and studied in relation to their action on brain health. The next subsections broadly classify NTs into canonical (the small molecules that are widely accepted as NTs) and noncanonical (neuroactive compounds that have recently been classified as NTs that remain controversial).

### 2.1. Canonical Neurotransmitters

**Figure 1:**
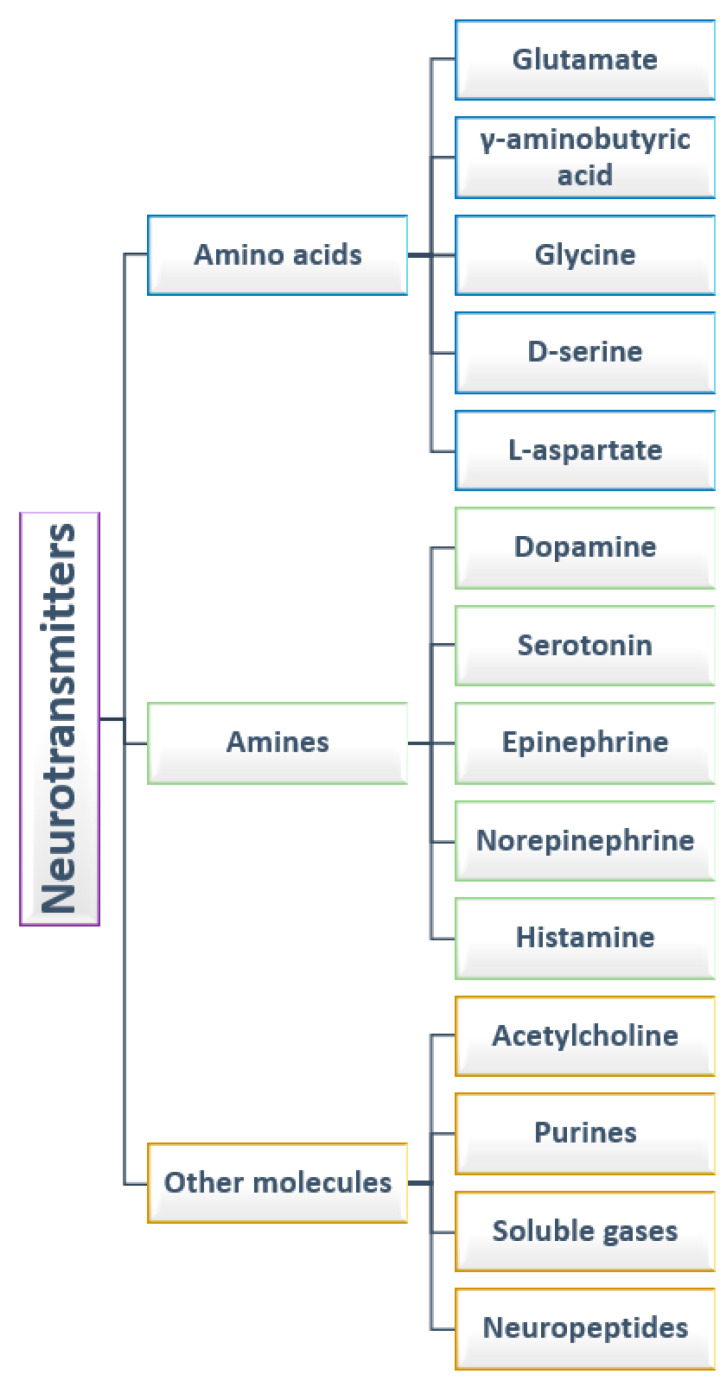
Classification of neurotransmitters.

There have been identified various types of NTs, with different functions produced in different regions of the brain [9]. From the point of view of their chemical nature, NTs are commonly divided into amino acids, amines, and other molecules. While depending on their activities, they can be of two types: positive and negative NTs or central and peripheral NTs [1]. The first classification is considered in the following subsections, as visually summarized in Figure 1.

#### 2.1.1. Amino Acids

Amongst NTs, amino acids represent a very important class of chemical messengers, having significant roles in the CNS [8]. The α-amino acids, such as glutamate and glycine, and γ-amino acids, such as γ-aminobutyric acid (GABA), are involved in fundamental brain processes and the pathogenesis of several disorders (e.g., epilepsy, stroke, dementia) affecting normal brain functioning [5,10].

Glutamate is a predominant excitatory NT in the CNS, which can be produced from glutamine and represents the precursor of GABA [11,12,13]. Glutamate is liberated from presynaptic neurons into the synaptic cleft, which leads to the activation of N-methyl-d-aspartate (NMDA) and alpha-amino-3-hydroxy-5-methyl-4-isoxazolepropionic acid (AMPA) receptors that further mediate calcium and sodium influxes in postsynaptic neurons. An excess of glutamate may produce excessive Ca^2+^ influx in the postsynaptic neuron. This further leads to extreme neuronal firing and excitotoxicity, being potentially involved in neurologic conditions, such as multiple sclerosis (MS), amyotrophic lateral sclerosis (ALS), and Parkinson’s disease (PD) [14].

**Figure 2:**
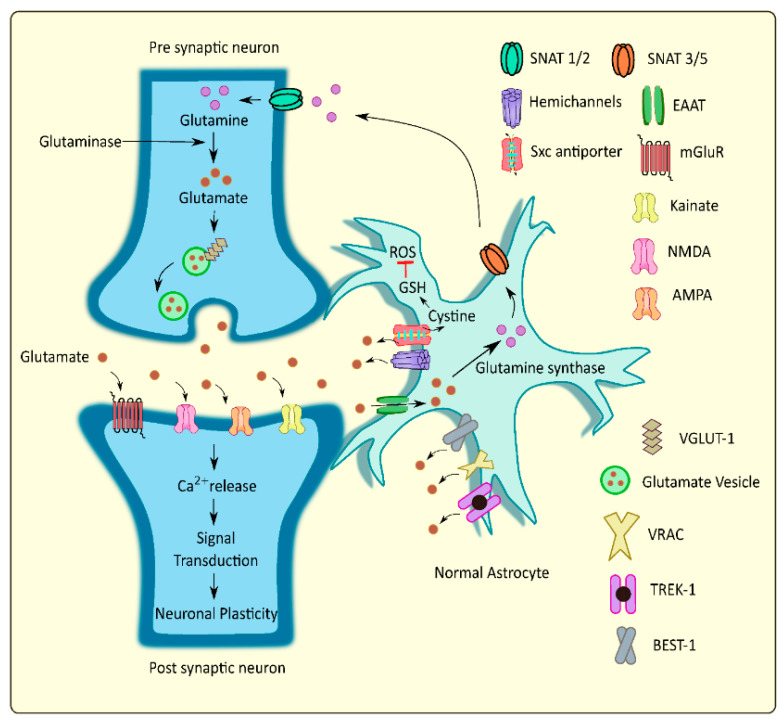
Schematic representation of glutamate homeostasis at the tripartite glutaminergic synapse. Reprinted from an open-access source [12]. Abbreviations: SNAT—sodium-coupled neutral amino acid transporter; Sxc antiporter—cystine/glutamate antiporter system xc; EAAT—excitatory amino acid transporter; NMDA—N-methyl-d-aspartate; AMPA—alpha-amino-3-hydroxy-5-methyl-4-isoxazolepropionic acid; VGLUT—vesicular glutamate transporter; VRAC—volume regulated anion channels; TREK—TWIK related potassium channel; BEST—bestrophin; ROS—reactive oxygen species; GSH—glutathione.

An essential role in maintaining proper extracellular levels of glutamate through release and uptake mechanisms is played by astrocytes. These cells mediate glutamate uptake and convert a part of it to glutamine, further transporting it to the presynaptic neurons, while certain amounts of the glutamate are released into the extracellular regions via different pathways. These processes regulate the glutamate homeostasis at the tripartite glutamatergic synapse [12] (Figure 2).

Glutamatergic neurotransmission is highly important in long-term potentiation, contributing to cognitive functions, such as learning and memory formation [11,13]. Moreover, it is also responsible for many motor, sensory, and autonomic activities [15]. Thus, it is crucial to maintain extracellular glutamate levels within a physiological range in order to ensure adequate neuronal transmission and viability. Being involved in such a wide range of functions, an imbalance of glutamate homeostasis can lead to significant neuropathological consequences [13,15,16]. Particularly, its disequilibrium has been linked to several neurological or neurodegenerative disorders, counting ALS [17,18], MS [19,20], Alzheimer’s disease (AD) [21,22,23,24], PD [15,25,26], Huntington’s disease (HD) [27], and epilepsy [24,28].

Another important amino acid NT is GABA, the main inhibitory NT in the brain that is formed through glutamate decarboxylase (EC 4.1.1.15) conversion of glutamate to GABA in interneurons [13,29] or is produced by commensal microorganisms from gut microbiota (e.g., *Bifidobacterium dentium*, *Lactobacillus brevis*) [30,31,32,33]. Nonetheless, studies revealed that GABA is initially an excitatory NT, as it induces a depolarization instead of hyperpolarization in various regions of the nervous system (e.g., neocortex, hippocampus, hypothalamus, cerebellum, spinal cord). This is caused by the existence of a higher chloride concentration in neurons during the early development of the human body, which is reflected in an outward instead of inward chloride flux. In contrast, there is a change of expression of sodium-potassium chloride co-transporters and the potassium chloride co-transporters in adults that modifies GABA action from excitatory to inhibitory [9]. Thus, it is widely accepted that low levels of GABA are responsible for the hyperexcitability of neurons [29]. This amino acid exerts its inhibitory activity by two types of specific receptors called GABAA (ionotropic) and GABAB (metabotropic) [13,34].

GABA neurons comprise a smaller fraction of the total neuronal population than glutamate. Nonetheless, maintaining the balance between inhibitory and excitatory transmission is imperative for normal brain functioning [35]. Hence, altered GABAergic neurotransmission has been associated with numerous CNS disorders, such as behavioral disorders, pain, and sleep [31], while stress and depression have been reported to disrupt the function of GABA [35]. Explicitly, impaired GABA homeostasis has been linked to various neurological disorders (e.g., autism spectrum disorders [36,37], schizophrenia [38], epilepsy [39,40,41]) and neurodegenerative diseases (e.g., MS [19,42,43], AD [44,45,46,47], PD [48,49,50], HD [51,52]).

In the spinal cord, the major inhibitory NT is glycine. This amino acid also acts as an NT in the brainstem and medulla, being a co-agonist with glutamate for NMDA receptors. Similar to GABA, glycine has an excitatory activity in early development, being employed in neuronal differentiation, proliferation, and connectivity. In adults, glycine has been observed to be involved in voluntary motor control, sensory processing, auditory, cardiovascular, and respiratory functions. [9,53].

Another amino acid, NT, is D-serine, a molecule released by glial cells whose functional role in a higher organism is relatively new. D-serine is produced from L-serine by serine racemase (EC 5.1.1.18), especially in the brain regions rich in NMDA-glutamate receptors [54,55]. In more detail, this NT is present in the rostral cerebral cortex, hippocampus, anterior olfactory nuclei, olfactory tubercule, corpus striatum, and amygdala, being particularly localized in the protoplasmic astrocytes of the grey matter that ensheath synapses [9].

One more amino acid to be included in this section is L-aspartate, whose role as an NT in the CNS has been subject to controversy. Discrepancies have been reported concerning the localization of this NT, as some studies proposed it to be an NT in the visual cortex and cerebellum, while others show that L-aspartate may be an NT and a neuropeptide-like modulator in the hippocampus. Moreover, the synaptic terminations that contain aspartate vesicles are co-localized with neurons containing glutamate and GABA vesicles. Thus, L-aspartate can play a role in both excitatory and inhibitory pathways.

#### 2.1.2. Amines

Monoamines are a representative group of NTs with clinical significance in motor functions, emotional responses, motivations, and behavioral functions [3,56]. These substances are synthesized from presynaptic neurons and bind to the corresponding receptors on the postsynaptic membrane in order to exert their functions. Moreover, the excess of monoamines remaining in the synaptic cleft is further degraded by monoamine oxidase (EC 1.4.3.4.) or catechol-O-methyltransferase (EC 2.1.1.6) or undergoes reuptake into the presynaptic terminal by monoamine transporters [11]. Severe neurological diseases (e.g., AD, PD, HD, schizophrenia) can occur when this equilibrium is dysregulated [56,57]. Thus, given their importance, the most representative NTs of this class are individually described in the next paragraphs.

Dopamine (4-(2-aminoethyl)-1,2-benzenediol) is one of the most important NTs in the mammalian nervous system as it seems to participate directly or indirectly in almost all physiological functions occurring in the CNS, thus being of great clinical relevance for motor functions and motivational behavior [58,59]. Dopamine is produced and released by dopaminergic neurons that are especially abundant in the substantia nigra pars compacta and in the ventral tegmental area [9,57]. Besides its role as an NT, dopamine is also involved in maintaining homeostasis and is a precursor for other catecholamines, such as norepinephrine and epinephrine [31,57]. As this amine NT participates in almost all centrally controlled events, ranging from motor control to cognition, its dysregulation may generate many psychiatric disorders (e.g., drug addiction, schizophrenia, PD, and HD) [9,57].

Another NT involved in regulating numerous physiological processes is serotonin (5-hydroxytryptamine). This amine NT is implicated in modulating sleep and wake states, gastrointestinal secretion and peristalsis, respiration, vasoconstriction, behavior (e.g., feeding behavior, aggressive behavior, and mood/depression), and neurological function [9,31,60]. The vast majority of serotonin (~95%) in the body is produced by enterochromaffin cells of the gut with the aid of the tryptophan hydroxylase enzyme (EC 1.14.16.4) [61,62,63]. In the brainstem, serotonin is mainly produced by rostral and caudal groups of neurons in the raphe nuclei, which further project to the cerebral cortex, thalamus, hypothalamus, and basal ganglia, and to the brainstem and spinal cord, respectively. Moreover, serotonin was proved to directly affect other neurotransmitters. Specifically, serotonin inhibits dopamine release, modulates glutamate and GABA transmission, inhibits glutamate release in the frontal cortex, and enhances glutamate transmission in the prefrontal cortex [13].

Epinephrine and norepinephrine are two monoamine molecules with dual roles: NTs and hormones. As NTs, they are involved in the autonomic nervous system (also known as the “fight or flight” system), composed of the sympathetic and parasympathetic systems. Norepinephrine neurons are found in the locus coeruleus, from where they project to various regions of the brain, including the limbic system [9]. From the point of view of its functionality, norepinephrine has been reported to have roles in arousal and alertness in the waking state, sensory signal detection, regulation of emotions, memory, learning, and attention [31]. On the other hand, epinephrine neurons are localized in different regions of the brain, counting the lateral tegmental system and medulla. However, its role as an NT is poorly understood. Epinephrine has been noticed to have an impact on the fight-or-flight response through the increase of heart rate, vasodilatation, pupil dilatation, and blood sugar levels [9].

Histamine is a signaling molecule acting as an NT in the CNS, being engaged in various physiological functions. It is synthesized and released by histaminergic neurons from the tuberomammillary nucleus of the hypothalamus, which further project to other regions of the brain (e.g., amygdala, cerebral cortex, substantia nigra, striatum, thalamus) and spinal cord. Studies have also linked the activity of this NT to disorders, such as AD and schizophrenia [9].

#### 2.1.3. Other Molecules

In addition to the above-presented substances, other molecules have also been identified as NTs. One of the most studied such molecules is acetylcholine, the first substance to be characterized and identified as a neurotransmitter in the peripheral nervous system. In the peripheral nervous system, it is released by the post-ganglion neurons in the parasympathetic system, being responsible for muscle contraction in the neuromuscular system. While, in the CNS, acetylcholine plays an essential role in consciousness, being associated with attention, learning, memory, consciousness, sleep, and voluntary movement control. Cholinergic neurons are localized in several brain and brain stem structures, including the striatum, cranial nerves, and vestibular nuclei [8,9]. From the cholinergic presynaptic neurons, acetylcholine is transported to synaptic vesicles via vesicular acetylcholine transporters, and, after the depolarization of neurons, it is released into the synaptic cleft. Further, it enables neurotransmission by binding to acetylcholine receptors [11].

Being a neuromodulatory agent in many areas of the forebrain, acetylcholine impacts numerous cognitive and motor functions through cortical and subcortical transmission in the cortico-striato-thalamocortical circuits [13]. Thus, its imbalances result in neurologic conditions, including AD, PD, HD, schizophrenia, myasthenia gravis, and other behavioral, learning, attention, memory, and sleep disorders [3,8,60].

Among other molecules recognized as NTs, there can be enumerated purines, such as adenosine triphosphate (ATP) [64], soluble gases (known as gasotransmitters), such as carbon monoxide (CO) [65,66], nitric oxide (NO) [65,66], and hydrogen sulfide (H_2_S) [66], and various neuropeptides [8,9], including somatostatin [67], β-endorphins [68], vasopressin [69], neurotensin [70], substance P [70], and neuropeptide Y [71]. One representative example from each category was chosen and briefly discussed.

ATP is the “energy currency” of the cell, being a source of the readily releasable energy required for numerous essential processes in organisms and cells, including signaling (i.e., intracellular, purinergic, synaptic), active transport, muscle contraction, and DNA/RNA synthesis. As synaptic transmission is an energy-demanding process, ATP is required at the presynaptic terminal to regulate ion gradients that shuttle NTs into vesicles and prepare vesicle release through exocytosis [72]. In the CNS, ATP is recognized as an excitatory neurotransmitter in neuron synapses—its deficient release being linked with many dysfunctions, including brain injuries, strokes, Parkinson’s disease, and Alzheimer’s disease [73].

Among soluble gases, NO is one of the most studied NTs, being well-established as a major signaling molecule and regulator of synaptic plasticity. Moreover, NO was observed to influence D-serine biosynthesis. In more detail, serine racemase is physiologically nitrosylated, inhibiting enzymatic activity and lowering the conversion rate of L-serine to D-serine. NO is produced in response to NMDA transmission and may diffuse to cells generating D-serine as a way of feedback inhibition [74].

Neuropeptide Y is one of the most widely expressed NTs in the nervous system, being the most abundant peptide present in the mammalian brain. This neurochemical was noticed to be employed in various biological processes, counting cortical excitability, stress response, food intake, circadian rhythms, and cardiovascular function. Thus, its abnormal regulation raises concerns about developing a broad range of conditions, including neurological diseases, such as epilepsy [75].

### 2.2. Noncanonical Neurotransmitters

Besides the widely accepted NTs discussed above, several other moieties have started to gain ground as noncanonical neurotransmitters, being still under intense scientific research and debate. Particular focus has been driven to the investigation of exosomes, a subtype of small bilipid layer extracellular vesicles (EVs) [1]. Exosomes have been reported as long-range messengers employed in the regulation of growth and development that facilitate intercellular communication, modulate antigen presentation and inflammation, and promote various stages of tumorigenesis [76]. Exosomes serve as mediators or regulators of neurotransmission and are morphologically similar to synaptic vesicles, yet they are released by their parent cells into extracellular spaces [1].

**Figure 3:**
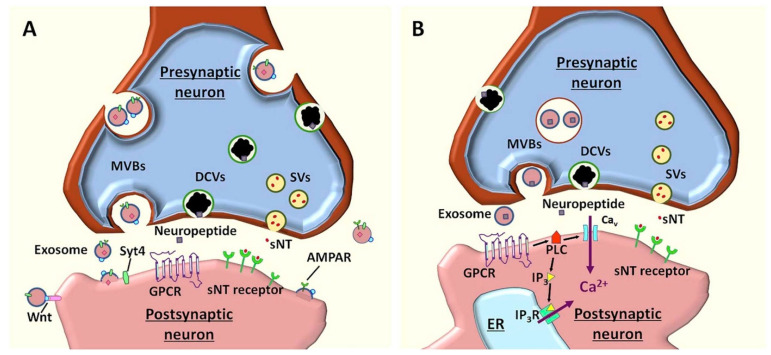
(**A**) Schematic representation of the function of neuronal exosomes as neuromodulators. In neurons, MVBs fuse with the plasma membrane without being restricted to the synaptic specialization and release exosomes into extracellular spaces, including the synaptic cleft. Through their various cargos, exosomes regulate synaptic plasticity. (**B**) The proposed models for neuronal exosomes as neurotransmitters. In response to action potentials, exosomes are released from the presynaptic neuron. As they carry neuropeptides and other ligands, exosomes activate GPCRs and downstream signaling cascade, leading to Ca^2+^ release from ER. Reprinted from an open-access source [1]. Abbreviations: AMPAR—alpha-amino-3-hydroxy-5-methyl-4-isoxazolepropionic acid receptor; DCV—dense core vesicles; ER—endoplasmic reticulum; GPCR—G protein-coupled receptors; IP_3_—inositol 1,4,5-triphosphate; IP_3_R—IP_3_ receptor; MVB—multivesicular bodies; PLC—phospholipase C; sNT—small neurotransmitter; SV—synaptic vesicles; Syt4—Synaptotagmin-4.

Concerning neurotransmission, exosomes were noted to modify presynaptic/postsynaptic signaling, control neurotransmitters release, support synapses, and enhance/suppress neurite growth and removal, axon regeneration, neurotransmitter production, and re-cycling. Exosomes contain various cargos, including Synaptotagmin-4 (Syt4), AMPA Receptor (AMPAR), and Wnt which regulate synaptic plasticity. In more detail, Syt4 trans-synaptic delivery augments synaptic growth and presynaptic release properties, AMPAR improves further neuronal excitability, whereas the Wnt ligand on neuronal exosomes activates Wnt signaling, thus regulating synaptic assembly, neurotransmitter release, and synapse remodeling (Figure 3A). In addition to these neuromodulatory activities, neuronal exosomes were also proposed as neurotransmitters due to a series of relevant characteristics. Namely, exosomes are released from presynaptic neurons in response to action potentials; they may transport neuropeptides, and other ligands that activate G protein-coupled receptors (GPCRs), thus activating phospholipase C (PLC) and generating inositol 1,4,5-triphosphate (IP_3_). Through the activation of GPCRs and downstream signaling cascade via multivesicular bodies (MVBs)-plasma membrane fusion controlled by SNARE molecules, neuronal exosomes induce Ca^2+^ release from the endoplasmic reticulum via IP_3_ receptors (IP_3_R) and Ca^2+^ influx through calcium channels (Figure 3B). The increase in Ca^2+^ intracellular levels is further reflected in inducing rapid responses in postsynaptic neurons, long-term changes in numbers of neuronal receptors, and long-term opening/closure of certain ion channels [1,77].

As exosomes are widely employed in normal communication in the CNS, nerve regeneration, synaptic function, and immune response, they have also been noticed to be involved in the propagation of neurodegenerative diseases, such as AD and PD [78]. Specifically, some exosomal proteins (e.g., Alix, Flotilin-1) have been noted to contribute to the pathogenesis of AD, as they were enriched in the Aβ plaques of diseased individuals, indicating the potential role of exosomes in forming Aβ deposits [76]. In relation to PD pathogenesis, exosomes were observed to contain higher amounts of α-synuclein than in healthy controls, readily transporting this protein from the cerebrospinal fluid to blood [79].

Another class of substances that have been recently started to be regarded as NTs is represented by steroids. It has been noticed that, besides their well-known activity as hormones, steroids also present neurotransmitter-like effects, such as signaling at the membrane and nucleus to regulate brain function, activating intracellular signaling cascades, gating membrane channels, increasing intracellular calcium release, and activating Src, MAPK, or phosphatidylinositol-3-kinase-AKT pathways [80].

One more small molecule gaining ground as potential NT is D-aspartic acid. This amino acid was found in the nervous tissues of several animals, including humans. Moreover, it has been observed to be present in the nervous system of rat and chicken embryos and human embryonic and adult brains, suggesting its involvement in the development of the nervous system and in adult neurological activity. Evidence pointing to the fact that D-aspartic acid may be an endogenous NT includes its presence in synaptic vesicles, synthesis occurrence in neurons via D-aspartate racemase conversion from L-aspartate, activity as a cell–cell signaling molecule, and elimination from postsynaptic neuron after its action [81].

## 3. Neurotransmitter Disorders of the CNS

As briefly mentioned in the above section, the variations in the levels, production, and metabolism of different NTs cause numerous diseases [8,12,82,83]. NT disorders are a group of inherited neurometabolic diseases owing to disturbances of NT metabolism, employing amino acids, cholinergic transmission, monoamines, purines, and other molecules (e.g., neuropeptides, ion channels, various neuromodulators) [84,85]. Nonetheless, these inborn errors are ultra-rare disorders. However, NT imbalances may also occur during one’s life due to impairment of neuronal receptors, intracellular signaling, vesicle release, or other synaptic abnormalities [86].

In this context, the next subsections aim to shed some light on the connection between the neurotransmitters’ alterations and the most common neurological and neurodegenerative disorders of the CNS.

### 3.1. Epilepsy

Epilepsy is a devastating neurological and systemic disorder characterized by seizures, resulting from a sudden and temporary synchronization of neuronal activity [87]. The altered NT signaling is considered a crucial feature of epileptic patients [29]. In particular, the imbalance between stimulant (i.e., glutamate) and suppressor (i.e., GABA) NTs significantly affects cell excitability. Given that glutamate is the main excitatory NT in the CNS, it has been widely agreed that glutamatergic hyperexcitation can provoke seizures [86,87,88,89].

In more detail, glutamate transporters, autoreceptors, and desensitization of postsynaptic receptors represent contributing factors to glutamatergic signals control. The direct activation of glutamate receptors can elicit seizures as the excessive recruitment of AMPA/NMDA receptors increases the permeability of the neuronal membrane to Na^+^, Ca^2+^, and K^+^ [2,86]. Notably, glutamate-induced Ca^2+^ overload is responsible for excitotoxicity, initiating cell death through apoptosis or necrosis in normal neuronal cells. Ca^2+^ may also accumulate in mitochondria, leading to its dysfunction, production of ROS and consequential oxidative stress, and eventual apoptotic neuronal death [29]. Thus, current anti-seizure medication aims to restore the NT balance by acting on ion channels, transporters, and receptors, thus exerting a symptomatic relief [87].

### 3.2. Multiple Sclerosis

Multiple sclerosis (MS) represents a chronic autoimmune-mediated inflammatory demyelinating disorder of unknown etiology, yet with a genetic predisposition and environmental influence [90,91,92]. MS is characterized by astroglial proliferation and neurodegeneration, with tissue damage being restricted to the CNS [93]. Moreover, it has been noticed that glutamate excitotoxicity is another feature of MS [20]. In addition, single nucleotide polymorphisms (SNPs) in glutamate transporters are associated with neurological disorders, including MS. In more detail, SNPs influence pathogenesis at the transcriptional level in the promoter regions as they may produce an altered binding of the transcription factors to the promoter, resulting in dysregulation of EAAT1/2 expression. This is reflected in high levels of extracellular glutamate and excitotoxicity, that further contribute to the onset and progression of the disease [94].

Another NT related to MS pathogenesis is GABA. Particularly, it was demonstrated that in patients with relapsing-remitting MS (RRMS) GABA+ levels are lower in the posterior cingulate cortex and left hippocampus as compared to controls. GABA level reduction may be caused by a decline in the efficacy of GABA synthesis or dysfunction in enzymes involved in glutamate-glutamine cycling. It was also suggested that dysfunctional GABAergic neurotransmission might contribute to cognitive impairment in RRMS patients. In more detail, properly working GABAergic interneurons provide recurrent inhibition to pyramidal neurons, stabilizing the inhibitory neural network and ensuring a normal cognitive performance; contrarily, when this system is impaired, the altered inhibitory signaling within cortical circuits produces a loss or deterioration in cognitive function [42]. Thus, it would be of good use to take these aspects into account to design effective and efficient MS medication.

In addition, genome screening studies revealed that many genes seem to be associated with MS, including human leucocyte antigens (HLA) classes I and II, T-cell receptor β, CTLA4, ICAM1, and SH2D2A [95].

### 3.3. Autism

Autism spectrum disorders (ASD) comprise a group of complex neurobehavioral and neurodevelopmental conditions primarily characterized by difficulties in social interaction and communication, restricted and repetitive patterns of behavior or interests, and altered sensory processing [96]. ASD is very heterogeneous and may have many underlying causes, with numerous neurochemical alterations being involved in its pathophysiology [97].

GABA and glutamate levels have been observed to be altered in children with ASD, creating an imbalance between excitatory and inhibitory mechanisms [96,98]. A recent study also suggested that prenatal exposure to a GABA_A_ receptor inhibitor leads to ASD-like behavior in offspring, being a potential underlying mechanism of these disorders [99]. Furthermore, glutamatergic projections to and from the various frontal sub-regions to the striatum are involved in regulating various compulsive behaviors, including stereotypy (i.e., repetitive motor behaviors) in ASD [100].

Moreover, NMDA and AMPA glutamate receptors are employed in ASD. Specifically, mutations of GRIN2A and GRIN2B genes have been associated with these disorders. Glutamatergic dysregulation and the development of autistic traits have also been linked with mutations in several genes (i.e., SHANK, NLGN3, NLGN4, and UBE3A) involved in synapse formation and maintenance [96].

**Figure 4:**
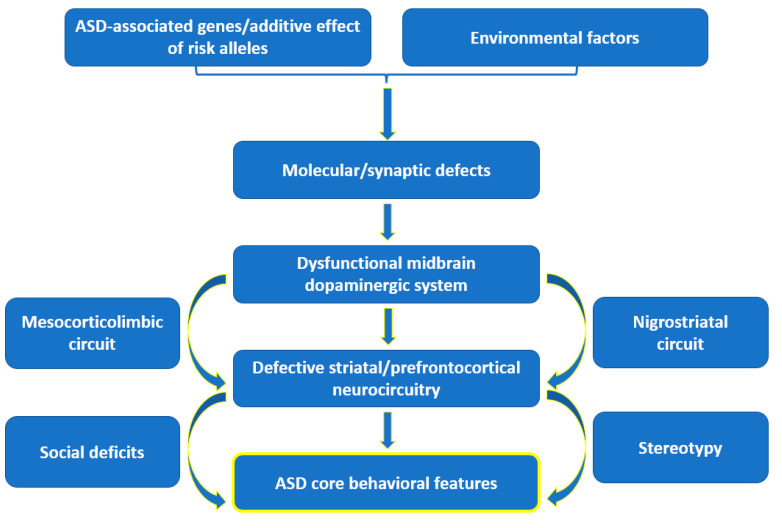
Schematic representation of the dopamine hypothesis. Created based on information from [102,103].

Abnormalities in monoamine NTs have also been correlated with ASD. Impaired dopamine, norepinephrine, and serotonin homeostasis are reflected in altered sleep patterns, mood, and the behavior of ASD patients [97,101]. Particularly, reduced dopamine release in the prefrontal cortex and reduced neural response in the nucleus accumbens have been observed in autistic subjects [96]. In addition, a recent hypothesis states that autistic behaviors arise from dysfunctions in the midbrain dopaminergic system as follows: a dysfunction of the mesocorticolimbic (MCL) circuit is responsible for social deficits, whereas dysfunction of the nigrostriatal (NS) circuit results in stereotyped behaviors (Figure 4). In more detail, signaling alterations in the MCL dopaminergic pathway leads to the hypoactivation of the reward system, impairing effort-based decision-making for rewards in autistic subjects. On the other hand, dysfunction of the NS pathway, which controls goal-directed motor behavior, was seen to entrap autistic individuals into loops of purposeless, stereotyped behavior patterns. Particularly, the severity of stereotypical behavior was noticed to be affected by polymorphisms of the dopamine 3 receptor, dopamine 4 receptor, and dopamine transporter genes [102].

Several other hypotheses have tackled the involvement of additional NTs in ASD, including acetylcholine, oxytocin, vasopressin, orexin, and endogenous opioids [96,97].

### 3.4. Alzheimer’s Disease

Alzheimer’s disease (AD) is a neurodegenerative disorder and the most common type of dementia, closely related to genetic factors and age. It predominantly affects neocortical regions and is characterized by progressively episodic memory loss, significant behavioral changes, and a very high mortality rate [90,104,105,106,107,108,109,110]. Nevertheless, the pathogenesis of AD remains elusive and mainly rests on the observed accumulations of amyloid beta into plaques and hyperphosphorylated tau protein aggregates. Other processes, such as changes in synaptic proteins, NT loss, oxidative damage, altered redox signaling, mitochondrial dysfunction, metabolic stresses, Ca^2+^ deregulation, inflammation, and cerebrovascular disease have been indicated as notable etiologies and potential mechanisms for neuronal and synaptic degeneration in AD [90,111,112,113].

There have been several hypotheses proposed for AD occurrence. The dominant model of AD pathogenesis is represented by the “amyloid cascade hypothesis”, which considers that the increased production of amyloidogenic Aβ42 peptide (or an increase in Aβ42:Aβ40 ratio) produces AD through a reduction of the number of synapses and neuronal cell death and degeneration [83,90,114]. Specifically, this cascade is triggered by the formation of insoluble toxic oligomers (AβO), which undergo structural modifications to form sheets that further create plaques and tangles, blocking proper neurotransmission [115].

An alternative possibility is the “calcium hypothesis of AD”, which states that the main driving force of neurodegeneration in AD is the imbalance in cellular calcium homeostasis. This can be explained by the fact that Aβ peptides form Ca^2+^—permeable pores in the cell membranes [83]. Thus, Aβ may induce an elevated calcium influx via L-type voltage-sensitive Ca^2+^ channels (VGCCs) [116]. More recently, it was also reported that Aβ oligomers suppress spontaneous synaptic activity by inhibition of P/Q-type VGCCs calcium currents [117]. Another source of intracellular calcium in AD patients may be considered the sustained activation of NMDA receptors, especially in the early disease stages, producing subsequent calcium overload and toxicity [83].

Moreover, deficiencies in various NTs have been noted to be responsible for neurodegenerative symptoms of AD: cognitive decline has been linked to cholinergic and glutamatergic deficits, synaptic plasticity deficits, and epileptiform symptoms have been associated with excitatory and inhibitory neurotransmission dyshomeostasis, while neuropsychiatric symptoms are closely related to monoamine neurotransmission modifications [11].

In more detail, an abnormal cholinergic system regulates and promotes changes in the metabolism of amyloid precursor protein and tau phosphorylation, triggering neurotoxicity, neuroinflammation, and neuronal death. Furthermore, as acetylcholine can regulate the normal cholinergic signal transduction (associated with learning and memory), its low levels in AD patients are no surprise, being manifested through damaged cholinergic signal transduction and cognitive impairment [11,104]. In particular, the cholinergic neurons forming the nucleus basalis of Meynert are specifically degenerated, leading to memory loss in individuals diagnosed with AD [115].

In addition, dopaminergic deficits were correlated with cognitive dysfunctions in AD patients, whereas the restoration of dopaminergic neurotransmission was noticed to rescue the pathologies and cognitive deficits. Hence, dopaminergic stimulation may be considered a potential therapeutic strategy. Moreover, dopaminergic dysfunction may also account for the behavioral changes in AD individuals, as this system is closely related to the brain reward [11]. Thus, it can be expected that, through the modulation of dopaminergic neurotransmission, the neuropsychiatric symptoms would be attenuated.

Likewise, extensive serotonergic denervation and serotonergic alteration were reported in AD individuals and were suggested to be involved in AD pathogenesis. Moreover, restoration of serotonergic function was proven to modulate behavioral and cognitive symptoms. Therefore, the serotonergic system represents a promising target for treating AD symptoms [11].

### 3.5. Parkinson’s Disease

Parkinson’s disease (PD) is a multifactorial and progressive neurodegenerative disease mainly characterized by the loss of dopaminergic neurons in substantia nigra pars compacta that project to the dorsal striatum. In more detail, the accumulation of misfolded and aggregated α-synuclein proteins leads to the formation of Lewis bodies, producing striatal dopamine deficiency reflected in a movement disorder [12,15,83,112,114,118,119,120]. Nonetheless, PD symptoms are not exclusively motor, as this disease is also associated with hyposomnia, dysautonomia, and sleep and psychiatric/cognitive disorders [90].

In addition to the extensive damage to dopamine-producing neurons, PD-related alterations have been noticed in various other NTs, including glutamate, GABA, serotonin, histamine, acetylcholine, and epinephrine [7].

**Figure 5:**
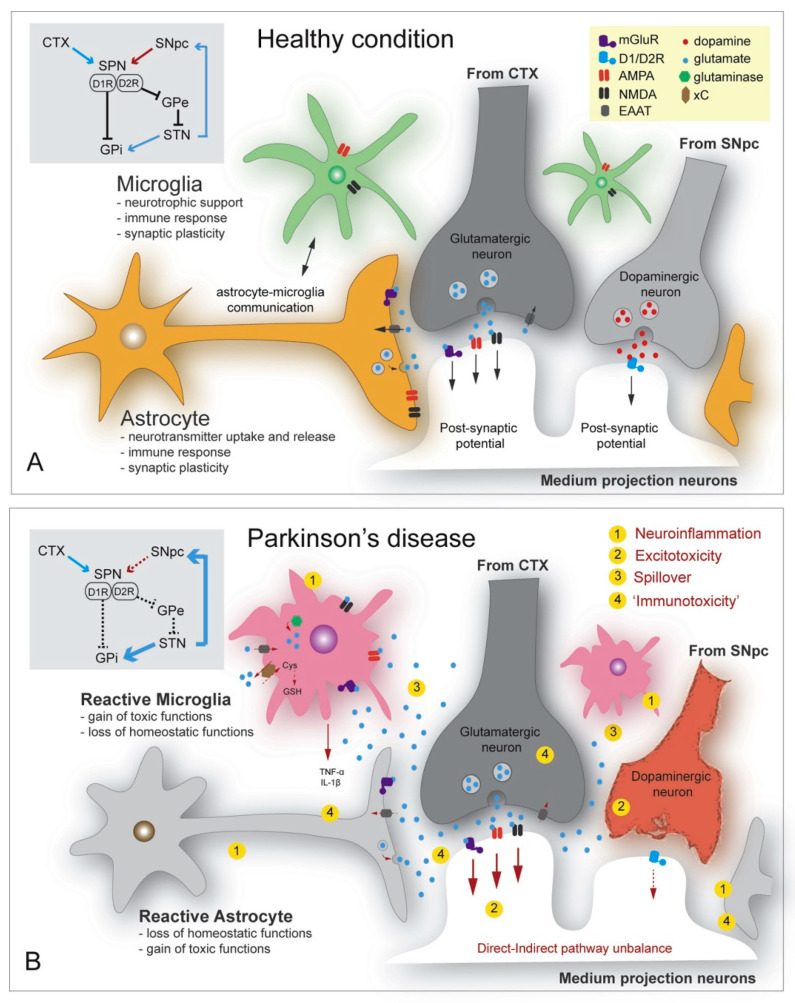
Glutamate and dopamine-related striatal events—focus on astrocytes and microglia functions in (**A**) healthy state, (**B**) Parkinson’s disease (PD) condition. Grey box: fronto-basal circuits involved in the modulation of voluntary movements and impaired connectivity caused by dopamine degeneration in PD. Reprinted from an open-access source [15]. Abbreviations: AMPA—alpha-amino-3-hydroxy-5-methyl-4-isoxazolepropionic acid; CTX—cerebral cortex; Cys—cysteine; EEAT—excitatory amino acid transporter; GPe—external segment of the globus pallidus; GPi—internal segment of the globus pallidus; GSH—glutathione; mGluR—metabotropic glutamate receptor; NMDA—N-methyl-D-aspartate; SNpc—substantia nigra pars compacta; SPN—spiny projection neuron; STN—subthalamic nucleus; xC—cysteine–glutamate exchange system.

In particular, dysregulation of glutamate homeostasis in the striatum is considered an emerging key feature of PD pathology [15]. More specifically, inflammation induces astrocytic glutamate excitotoxicity that modifies glutamate transporters and receptor expression. The accumulation of α-synuclein was also noted to enhance the Ca^2+^ depolarization-dependent release of presynaptic glutamate, further activating the extra-synaptic NMDA receptors and producing neuronal damage. The increase in glutamate levels activates the AMPA receptors, further upregulating glutamate release. Moreover, α-synuclein mobilizes glutamate vesicles from the pool of reserves and produces mGluR5 overexcitation [12]. These mechanisms are visually represented in Figure 5, treated as a comparison between healthy and diseased states.

### 3.6. Huntington’s Disease

Huntington’s disease represents a monogenic autosomal dominant inherited neurodegenerative brain disorder manifested through motor and cognitive disturbances. It is caused by the CAG trinucleotide repeat expansion in the first exon of the huntingtin (Htt) gene that results in polyglutamine expansions and pathogenic aggregation initially in neurons of striatal and cortical motor and prefrontal areas [83,113,119,121].

In HD, abnormal body movements are caused by an imbalance in the activity equilibrium of direct and indirect pathways. Particularly, the striatal levels of various NTs are altered as follows: dopamine—increased in the early stages and decreased in the late stages; GABA—decreased; glutamate—increased; adenosine—decreased; and acetylcholine—increased [122]. The most profound neurodegeneration caused by HD takes place in the caudate and putamen due to the high levels of dopaminergic innervation and dopamine receptors. In the early stages of HD, hyperkinetic movements are caused by heightened thalamocortical glutamatergic signaling driven by the loss of neurons in the indirect pathway. In contrast, in the advanced disease stages, when both direct and indirect pathways are affected, hypokinesia occurs. Hence, studies concluded that there is a synergistic action between the dopamine and glutamate signaling pathways that can enhance toxicity through D1 receptor activation [123].

### 3.7. Schizophrenia

Schizophrenia is a severe heterogeneous multifactorial neurodevelopmental disorder with unknown etiology, generally diagnosed in adolescence [38,124]. It is characterized by a combination of positive, negative, and cognitive symptoms, out of which the positive ones (e.g., hallucinations, delusions) are the most representative of this disease. However, cognitive issues, including deficits in working memory, executive function, learning and long-term memory, visual/auditory perception, and attention, are generally present prior to the onset of psychosis, being the best predictors of long-functional outcome [125].

Some of the cognitive symptoms can be explained by the primary deficits in NMDAR glutamate synaptic signaling. Particularly, deficits from layer 3 pyramidal neurons in the prefrontal cortex are thought to cause impaired executive cognitive functions, undermining recurrent excitation and the maintenance of information in working memory [126].

Alternatively, dysregulations of GABA neurotransmission represent a potential explanation for working memory deficits. Specifically, working memory function is associated with gamma frequency oscillations in the prefrontal cortex, which have been noted to have lower power in individuals with schizophrenia. As GABA neurons are considered generators of gamma oscillations via the pyramidal interneuron network gamma model, their dysfunction can be correlated with reduced gamma oscillatory power and working memory deficits [125].

In schizophrenia, reduced GABAergic neurotransmission has also been associated with increased dopamine synthesis [38]. Moreover, presynaptic and postsynaptic dopaminergic abnormalities have been linked to the onset of mental illness, with the dysregulation of the dopaminergic system being one of the main culprits in the etiology of schizophrenia [124].

### 3.8. Depression

Depression represents another highly heterogeneous disorder that, besides behavioral factors, has been linked with several biological mechanisms, counting inflammatory response, hypothalamic-pituitary-adrenal axis dysregulation, sympathetic and parasympathetic nerve systems imbalance, and endothelial dysfunction with platelet activation [127,128].

Neurobiological studies unraveled that depression is associated with the atrophy of neurons in cortical and limbic brain regions and altered brain connectivity and network function. These alterations are caused by structural, functional, and neurochemical deficits, with particular involvement of GABA and glutamate systems dysfunction [35].

A different hypothesis focuses on the connection between depression and low levels of monoamine NTs, as depressed subjects have been noted to exhibit reduced concentrations of serotonin, dopamine, and norepinephrine that can be increased by the administration of antidepressant drugs. Nonetheless, studies concerning monoamine levels lead to contradictory results, whereas GABAergic and catecholaminergic pathways had better diagnostic values [127,129].

### 3.9. Amyotrophic Lateral Sclerosis

Amyotrophic lateral sclerosis (ALS) represents a progressive neurodegenerative disease with complex pathogenesis [18]. It was initially considered a pure motor neuron disease, but it has been since recognized as a multisystem neurodegenerative disorder, with disease heterogeneity at the clinical, genetic, and neuropathological levels [130]. ALS encompasses varied manifestations, including motor neuron degeneration, muscle wasting, paralysis, and severe metabolism deregulation [113].

The mechanisms behind ALS occurrence comprise reactive oxygen species (ROS)-associated oxidative stress, mitochondrial dysfunction, impaired homeostasis, axonal and vesicular trafficking dysregulation, glutamate excitotoxicity, proteostatic impairments, altered RNA metabolism and/or processing, low Ca^2+^ buffering capacity, a high number of AMPA receptors in motor neurons, neuroinflammation, and neurotrophins depletion [12,14,114]. There is also a genetic component of ALS risk, as mutations in SOD1 have been observed in ~2% of ALS cases. Other common genetic risk factors that increase the probability of ALS occurrence are an intermediate expansion of the CAG trinucleotide repeat in the ataxin-2 (ATXN2) gene, variants in elongator complex protein 3 (ELP3), survival motor neuron protein (SMN1) copy number variation, and insertion or deletion mutations in the neurofilament heavy polypeptide (NEFH) gene [131].

As ALS patients exhibit accumulated glutamate levels and associated neurotoxicity [12], efforts have been made to create treatments based on decreasing the NT’s concentration. Particularly, riluzole, a drug with antiglutamatergic effects, is the only approved disease-modifying drug in most European countries. This treatment is reported to prolong patient survival by 3 to 6 months, with the drawback of side effects, such as nausea, diarrhea, fatigue, dizziness, and liver problems [130].

## 4. Neurotransmitters Detection

Chemically diagnosing brain disorders represents an extremely challenging task. Nonetheless, the monitoring and detection of NTs at early disease stages is highly needed to avoid risk factors in their associated disorders. Brain disorders’ biomarker detection is mainly hampered by the blood–brain barrier (which maintains strict and different chemical climates between the brain and the periphery) and difficulty to probe the chemistry, i.e., neurotransmission, of an intact brain in vivo [8,60,90].

Consequently, increasing research interest has been noted in overcoming these challenges, and, in recent years, developments have been reported in designing and optimizing tools for the direct detection of chemical biomarkers involved in neurological disorders [60,90,132,133,134]. The diagnosis and detection of NTs in direct and biological samples can be performed using various tools, including electrochemical methods, fluorescence resonance energy transfer, chemiluminescence, various combinations of chromatography, mass spectrometry, and capillary electrophoresis (i.e., GC, HPLC, GC-MS, LC-MS, CE-MS), surface-enhanced Raman spectroscopy, and NIR-based biosensing, and microdialysis methods [8,135,136,137,138,139].

Noteworthy advances have been observed in nanomaterial-based working systems, especially due to the advantages of these nanosized materials in terms of sensitivity, selectivity, and lower detection limits. Thus, various carbon-based, metal-based, metal-oxide-based, polymer-based, and enzyme-based nanosensors could hold promise for performant detection and monitoring of different NTs [3,8,140,141,142,143,144,145]. A selection of newly developed nanomaterial-based NT detection systems has been gathered in Table 1.

## 5. Modulation of Neurotransmitters and Neurotransmitter Transporters as a Therapeutic Strategy

After accurately detecting the levels of NTs and assessing the risk of developing associated neurological disorders, it is of vital importance to adjust them towards normal values. In this respect, a potential therapeutic strategy is considered for the modulation of neurotransmitter transporters (NTT) employed in fine-tuning brain NT homeostasis [172]. Specifically, plasma membrane NTTs keep extracellular concentrations at a certain level by facilitating NT transport into the cytosol, limiting receptor binding, and activating downstream signaling pathways [173]. Hence, NTTs represent attractive pharmacological targets for the treatment of relevant neurological and neuropsychiatric diseases [172].

For instance, glutamate-aspartate transporter (GLAST) and glutamate transporter-1 (GLT-1) and their human homologs, excitatory amino acid transporter 1 (EAAT1) and 2 (EAAT2) are primarily expressed in astrocytes and are responsible for maintaining optimal extracellular glutamic levels, preventing accumulation in the synaptic cleft, and avoiding excitotoxicity [94,173]. The expression and function of these NTTs can be enhanced by the administration of pharmacological agents, such as β-lactam antibiotics, estrogen/selective estrogen receptor modulators, growth factors, histone deacetylase inhibitors, and translational activators [94]. Alternatively, glutamatergic signaling can be modulated via mGluR agonists, as increased mGluR3 activation would inhibit L-type calcium channels, resulting in reduced amounts of glutamate in the synaptic cleft [174].

Interesting possibilities can be envisaged from the modulation of monoamine transporters (MATs) which are employed in the reuptake of dopamine, serotonin, and norepinephrine from extra-neuronal regions and preservation of NT homeostasis. Thus, MATs are relevant targets for many compounds, including antidepressants, substances of abuse, and medicines for neuropsychiatric and neurodegenerative disorders [175]. Caffeine has also been reported as a valuable modulator for NT systems in mesocorticolimbic brain regions, holding promise for attenuating certain neurological diseases through modulating dopaminergic signaling. Particularly, caffeine was observed to protect against dopaminergic neuronal loss, being a potential therapeutic agent against Parkinson’s disease [176].

Caffeine was also noted to influence the activity of glutamatergic and GABAergic neurons. Specifically, caffeine exposure may increase glutamate levels and modulate glutamatergic receptors and transporters, while it can induce various effects on GABAergic systems, including GABAergic receptors, towards improving neurobehavioral disorders. Nonetheless, when creating caffeine-based treatments, it should be kept in mind that, at high doses, this substance may induce neurotoxicity, negative neurobehavioral effects, and undesirable health responses in other systems, such as cardiovascular, skeletal, and muscular systems [176].

Potential modulation approaches also arise from the ingestion of prebiotics and probiotics, as specific microbes in the gastrointestinal tract have been noted to regulate NT levels via the gut-brain axis [177]. For instance, ingestion of probiotic *Lactobacillus rhamnosus* has been demonstrated to increase GABA_B1b_ mRNA in cortical regions and reduce its expression in the hippocampus, amygdala, and locus coeruleus; increase GABA_Aα2_ mRNA expression in the hippocampus, and reduce its levels in the prefrontal cortex and amygdala. Thus, this bacterial strain can be considered an adjuvant therapeutic agent in stress-related disorders, including anxiety and depression [178]. On the other hand, consumption of prebiotic chitooligosaccharides was reported to strongly inhibit acetylcholinesterase (EC 3.1.1.7), being, therefore, a beneficial material for preventing or treating AD [179].

It is noteworthy to mention that certain drugs can mimic NTs and alter neurotransmission by interacting with specialized receptors and transporters. For example, heroin resembles the brain’s natural opioids but stimulates many more receptors more strongly, leading to massive amplification of opioid receptor activity; marijuana mimics cannabinoid NTs; nicotine attaches to acetylcholine receptors and indirectly induces an increase in glutamate levels; cocaine attaches to the dopamine transporter, blocking this NT to re-enter the neuron and leading to a much greater dopamine impact on the receiving neurons than occurs naturally manifested through euphoria; cocaine also alters norepinephrine and glutamate systems, producing stimulant effects. Consequently, drug abuse leads to numerous physiological dysfunctions [180].

## 6. Conclusions

In summary, neurotransmitters are chemical moieties that can carry and amplify signals, ensuring information transmission throughout the nervous system. As they comprise a wide range of molecules (e.g., amino acids, amines, purines, soluble gases, neuropeptides) and are involved in numerous functions (e.g., emotions, thoughts, memories, movements, learning, sleep patterns, behavior, alertness, arousal, vasoconstriction, respiration), neurotransmitters are essential factors in maintaining brain health. Consequently, disturbed neurotransmitters’ homeostasis and/or impaired neurotransmission results in severe diseases that significantly impact patients’ lives and the global health system.

To recapitulate, altered levels of neurotransmitters, such as glutamate, GABA, dopamine, serotonin, norepinephrine, histamine, and acetylcholine, were noticed to be involved in the pathophysiology of a long list of diseases, including autism spectrum disorders, schizophrenia, epilepsy, multiple sclerosis, amyotrophic lateral sclerosis, Parkinson’s disease, Huntington’s disease, Alzheimer’s disease, drug addiction, depression, and sleep disorders. Hence, monitoring and detecting NTs at early disease stages is mandatory to avoid complications of these associated disorders. Nonetheless, chemically diagnosing brain disorders represents a highly challenging task, and, despite recent advancements in designing nanotechnology-based NT sensors, there is still room for improvement of current detection methods. Moreover, further in-depth research is needed to investigate neurotransmitters’ complex mechanisms of action and develop strategies for modulating their levels toward maintaining homeostasis.

To conclude, neurotransmitters are intensively investigated, as they are key factors in many neurological and neurodegenerative disorders. Thus, it can be expected that, by conducting interdisciplinary research studies, the elucidation of neurotransmitters’ roles would help design efficient treatments for restoring the quality of life of millions of patients worldwide.

## Figures and Tables

**Table 1:** Examples of nanomaterial-based NT detection systems.

Detection System	Detected NT	Performance Indicators	Ref.
Glutamate oxidase entrapped in a chitosan matrix cast onto the microelectrode surface (i.e., platinum wire covered with poly-o-phenylenediamine) and coated with ascorbate oxidase	Glutamate	Sensitivity: 0.097 ± 0.001 nA μM^−1^ Linearity range: 5–150 μM Detection limit: 0.044 μM	[146]
Monolithic platform based on carbon-nanotube field-effect transistors	Glutamate	Linearity range: 250–500 μM Detection limit: 3 μM	[147]
Perovskite nickelate-Nafion (i.e., polymeric ion-permeable membrane) heterostructure	Glutamate	Sensitivity: 0.327 ± 0.07 nA μM^−1^ mm^−2^ Linearity range: 1–700 μM Detection limit: 16 nM	[148]
Reduced graphene oxide-based field-effect transistor biosensor functionalized with mGluR	Glutamate	Linearity range: 1 fM–100 pM Detection limit: 1 fM	[149]
Enzyme-free electrochemical sensor based on graphene oxide modified gold electrode	GABA	Linearity range: 250 nM–100 μM Detection limit: 98 nM	[150]
nanoITIES (interface between two immiscible electrolyte solutions) pipet electrodes	GABA	Linearity range: 0.25–1.0 mM Detection limit: 22.4 μM	[151]
Gold nanoparticles-zinc oxide nanocone arrays/graphene foam electrode	Dopamine	Sensitivity: 4.36 μA mM^−1^ Detection limit: 0.04 μM	[152]
Label-free luminescent NaGdF_4_:Tb nanoparticles	Dopamine	Linearity range: 0–10 μM Detection limit: ~30 nM	[153]
Supramolecular β-cyclodextrin functionalized gold nanoclusters	Dopamine	Linearity range: 100.0 nM–80.0 μM Detection limit: 20.0 nM	[154]
Graphite screen-printed electrodes modified by a nanocomposite made of polyaniline and gold nanoparticles	Dopamine	Linearity range: 1–100 μM Detection limit: 0.86 μM	[155]
Nanocomposite platform based on graphene oxide/chitosan modified screen-printed electrode	Serotonin	Sensitivity: 0.05 μA mM^−1^ Detection range: 10 nM–100 μM Detection limit: 3.2 nM	[156]
Gold-nanorattles-reduced graphene oxide nanocomposite coated onto the gold nanoparticles deposited glassy carbon electrode (GCE)	Serotonin	Linear dynamic range: 3 × 10^−6^–1 × 10^−3^ M Detection limit: 3.87 (±0.02) × 10^−7^ M	[157]
Graphite-paste electrode modified with nanoparticles (i.e., Fe_3_O_4_@Au@SiO_2_) coated with molecularly imprinted polymer	Serotonin	Linearity range: 0.01–1000 μM Detection limit: 0.002 μM	[158]
GCE coated with a biofilm of graphite, nanodiamonds, and gold nanoparticles anchored in casein	Serotonin	Sensitivity: 0.18 μA mM^−1^ Linear dynamic range: 0.3–3.0 μM Detection limit: 0.1 μM	[159]
Platinum nanoparticles coated with molecularly imprinted silica drop-cast onto a GCE	Serotonin	Linearity range: 0.05–80 μM Detection limit: 0.02 μM	[160]
Laccase modified GCE coated with graphene quantum dots	Epinephrine	Sensitivity: 2.9 μA mM^−1^ cm^−2^ Linearity range: 1–120 × 10^−6^ M Detection limit: 83 nM	[161]
Tetrahexahedral gold-palladium core-shell nanocrystals on reduced graphene oxide nanosheets	Epinephrine	Linear detection range: 0.001–1000 μM Detection limit: 0.0012 μM	[162]
GCE modified with chemically reduced graphene oxide nanosheets	Epinephrine	Two different linearity ranges: 10–300 and 400–1300 μM Detection limit 1.6 μM	[163]
Graphite screen-printed electrode modified with a nanocomposite of magnetic Fe_3_O_4_@SiO_2_ nanoparticles and carbon nanotubes	Norepinephrine	Linearity range: 0.5–400 μM Detection limit: 0.2 μM	[164]
GCE modified with carbon nanotubes and magnetic nanoparticles of cobalt ferrite	Norepinephrine	Linearity range: 0.16–1.91 mM Detection limit: 0.76 μM	[165]
Copper-palladium core-shell nanostructures on pencil graphite substrate	Histamine	Sensitivity: 0.082 μA μM^−1^ cm^−2^ Detection limit: 3.2 ± 0.1 nM	[166]
Nickel-based metal-organic framework crystals and multi-walled carbon nanotubes modified GCE	Histamine	Sensitivity: 0.19 μA μM^−1^ Linearity range: 1.00–160.00 μM Detection limit: 0.41 μM	[167]
Fiber-optic surface plasmon resonance (SPR)-based biosensor covered with multilayers of silver metal and tantalum (V) oxide nanoflakes functionalized with acetylcholinesterase enzyme	Acetylcholine	Sensitivity: 8.709 nm/μM Detection limit: 38 nM	[168]
Acetylcholinesterase and choline esterase co-immobilized on platinum nanoparticles and metallic organic framework modified gold electrode	Acetylcholine	Linearity range: 0.01–500 μM Detection limit: 0.01 μM	[169]
Acetylcholinesterase and choline esterase co-immobilized over a gold electrode coated with a nanocomposite layer of multi-walled carbon nanotubes and reduced graphene oxide	Acetylcholine	Linearity range: 0.1–100 μM Detection limit: 0.1 μM	[170]
Enzyme-free electrochemical sensor based on spinel-type copper cobaltite nanoplates	Acetylcholine	Linear dynamic range: 0.2–3500 μM Detection limit: 30 nM	[171]

## Data Availability

Not applicable.
